# Homocysteine is associated with higher risks of ischemic stroke: A systematic review and meta-analysis

**DOI:** 10.1371/journal.pone.0276087

**Published:** 2022-10-13

**Authors:** Nícollas Nunes Rabelo, João Paulo Mota Telles, Leonardo Zumerkorn Pipek, Rafaela Farias Vidigal Nascimento, Rodrigo Coimbra de Gusmão, Manoel Jacobsen Teixeira, Eberval Gadelha Figueiredo

**Affiliations:** 1 Department of Neurosurgery, University of Sao Paulo, Sao Paulo, Brazil; 2 Faculdade de Medicina do ABC - Centro Universitário Saúde ABC, Santo André, São Paulo, Brazil; The University of Mississippi Medical Center, UNITED STATES

## Abstract

**Background:**

High levels of homocysteine (Hct) have been associated with great risks of ischemic stroke. However, some controversy still exists. We performed a systematic review and meta-analysis to compare the levels of Hct between patients with ischemic stroke and controls.

**Methods:**

We performed a systematic literature search for articles reporting Hct levels of patients with occurrence of ischemic stroke. We employed a random-effects inverse-variance weighted meta-analytical approach in order to pool standardized mean differences, with estimation of τ^2^ through the DerSimonian-Laird method.

**Results:**

The initial search yielded 1361 studies. After careful analysis of abstracts and full texts, the meta-analysis included data from 38 studies, which involved almost 16 000 stroke events. However, only 13 studies reported means and standard deviations for cases and controls, and therefore were used in the meta-analysis. Those studies presented data from 5002 patients with stroke and 4945 controls. Standardized mean difference was 1.67 (95% CI 1.00–2.25, P < 0.01), indicating that Hct levels were significantly larger in patients with ischemic stroke compared to controls. Between-study heterogeneity was very large (*I*^*2*^ = 99%), particularly because three studies showed significantly large mean differences.

**Conclusion:**

This meta-analysis shows that patients with ischemic stroke have higher levels of Hct compared to controls. Whether this is a modifiable risk factor remains to be assessed through larger prospective cohorts.

## Introduction

As a demethylated by-product of methionine, homocysteine (Hct) is considered a toxic amino acid. It is usually removed by methionine synthase in a remethylation process that uses methylcobalamin as a co-factor and 5-methyl tetrahydrofolate as a methyl donor. In the brain, Hct catabolism heavily relies on its remethylation to methionine using methylcobalamin and folate.

Since B vitamins namely vitamin B6, vitamin B12, and folate are considered as critical co-factors during catabolism process of the Hct, an increased Hct concentration (iHct) can signal a lack or deficiency of vitamin B [1]. The Hct-methionine metabolism plays a significant role especially providing a methyl donor known as S-adenosylmethionine (SAM) which is used to perform several biological reactions.

Ultimately, iHct is known to cause several damages to major brain vessels either by altering the methylation potential (SAM/SAH) or acting independently, leading to ischemic stroke [2].

Ischemic stroke has been a leading cause of mortality and morbidity across the globe, causing a significant burden to patients and families [3, 4]. There are several known factors elevating the risk of ischemic stroke, which include transient ischemic attack (TIA), arterial diseases, atrial fibrillation, improper diet and/or obesity and physical inactivity [5]. Notably, less than half of the different types of strokes can be linked to recognized causal risk factors [6, 7].

Lately, attention has been given to Hct and its role in stroke incidences or events. Hct is a type of amino acid that contains sulfur that is considered a causal risk factor for vascular diseases such as stroke. Furthermore, available empirical evidence shows that there is a positive correlation between iHct and ischemic stroke, which has helped develop practical procedures for screening and treating iHct as a modifiable risk factor [7, 8]. Nevertheless, there are still some controversies regarding the causal role of iHct in stroke, especially due to failure of vitamin B-supplementing trials in preventing strokes [9].

Apart from a meta-analysis by Zhang et al. (2020) [10] particularly focused on the Chinese population, to the best of our knowledge, the last broad scope meta-analysis on the subject was published in 2014 by He and colleagues [11]. Therefore, we carried out a systematic review and meta-analysis to compare the levels of Hct between patients with ischemic stroke and controls. This systematic review included the largest number of studies until now, with a significant number of different populations, almost 16 000 events and a analysis based on stroke subtypes.

## Methods

### Literature search strategy and study design

**Fig 1:**
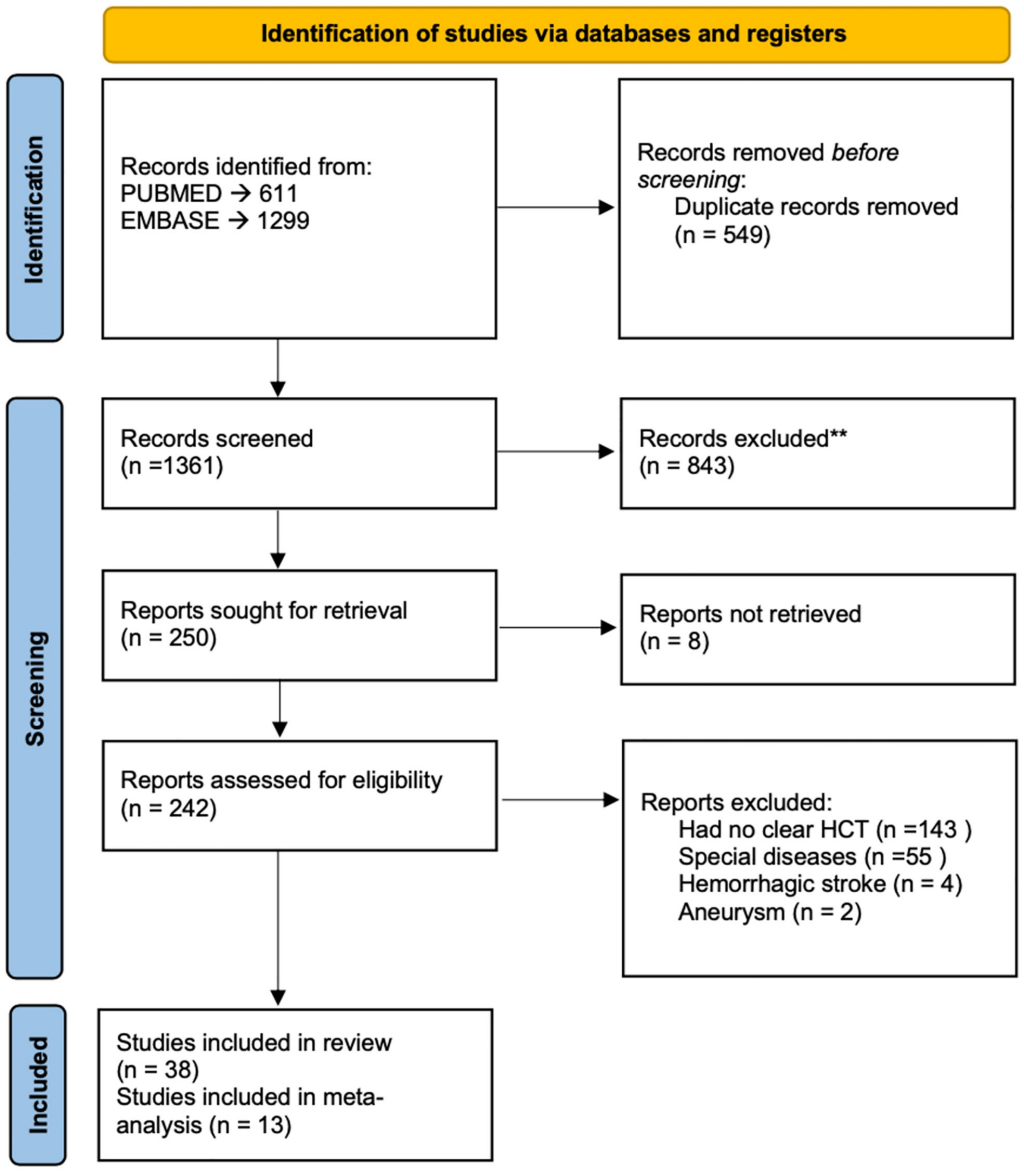
Flow diagram. PRISMA flow diagram of literature search strategy.

This systematic review and meta-analysis was conducted according to Preferred Reporting Items for Systematic Review and Meta-Analysis (PRISMA) [12]. The literature search was conducted using EMBASE and PubMed. We considered all articles up to August 13, 2022 that assessed the link between homocysteine and ischemic stroke. Keywords used include “Plasma homocysteine”, “Homocysteine”, “Hyperhomocysteinemia”, and “Ischemic Stroke” [13–24]. Other relevant articles were searched through snowballing (Fig 1). Two authors independently extracted data and performed a quality assessment of the articles using the Newcastle-Ottawa Scale (NOS). Disagreements were solved by a third author.

The search strategy used was:

(“Plasma homocysteine”[All Fields] OR “Homocysteine”[All Fields] OR “Hyperhomocysteinemia”[All Fields]) AND “Ischemic Stroke”[All Fields]—PUBMED
(‘plasma homocysteine’ OR ‘homocysteine’/exp OR ‘homocysteine’ OR ‘hyperhomocysteinemia’/exp OR ‘hyperhomocysteinemia’) AND (‘ischemic stroke’/exp OR ‘ischemic stroke’)—EMBASE

### Inclusion and exclusion criteria

The inclusion criteria were articles published in English about Hct concentrations in patients with ischemic stroke and controls. Randomized controlled trials (RCTs) and cohort studies were included in our systematic review. Characteristics of included studies are shown in Table 1. Articles were excluded if they were reviews, meta-analyses, letters, case reports, and articles in which Hct levels were not reported [1].

**Table 1:** Studies reporting homocysteine levels in patients with ischemic stroke.

First Author	Year	Country	Design of study	Type of study	Detecting Method	Number of Patients With IS	Number of Control Group	IS Group—Hct, mmol/L, MEAN (SD)	Control–Hct, mmol/L, MEAN (SD)	Enrollment period	Note
Zhou [25]	2022	China	Prosp	Case-cohort	NI	3070	3070	14.1 (7.8)	13.4 (8.2)	2016–2018	Elevated tHcy is the second most important contributor and acts additively with SBP to increase the risk of the first stroke
Shademan [26]	2021	Turkey	Retro	Case-control	ELISA	120	120	16.1 (1.20)	13.2(0.82)	2019–2020	Homocysteine were significantly higher in IS patients than in the control group.
Hultdin [38]	2011	Sweden	Retro	NI	NI	321	397	12.8±5.6 mmol/L	12.7±7.7 mmol/L	1986–1999	Noticed association between iHct and HS, but not between iHct and IS.
Hogeveen [40]	2002	Netherland	Retro	Case-control	NI	11	94	8.6 (4.0–16.0) mmol/L	7.4 (4,8–18.0) mmol/L	1993–2000	iHct was associated to HS and IS in newborns
Hongli [42]	2006	China	Retro	Case-control	HPLC	126	NI	17.5 ±10.9 mmol/l	NI	NI	Included association between IS and iHct to IC stenoses. Didn´t include control group.
Verhoef [43]	1994	United States	Prosp	Case-series	NI	109	427	11.1±4.0 mmol/L	10.6±3.4 mmol/L	1982–1984	Noticed difference between groups without SS (p = 0.12)
Kelly [33]	2004	United States	Prosp	Case-series	FPA	156	118	10.1 mmol/L	10.33 mmol/L	2003	Used geometric mean to calculate Hct. Without SS
Chao-Zhi [44]	2015	China	Prosp	Case-series	Standard laboratory methods	226	NI	15.6 (12.2 ± 23.2) mmol/L	NI	2012–2013	Didn´t include control group.
Hyung-Min [45]	2014	South Korea	Retro	Case-control	NI	396	NI	11.4±4.7 mmol/L	NI	2013	Didn´t include control group.
Weikert [46]	2007	Germany	Retro	Case-control	NI	188	779	9.7 (8.4–11.6) mmol/L	8.4 (6.8–10.2) μmol/L	1994–1998	NI
Zhihong [47]	2015	China	Prosp	Case-series	Nephelometric technology	3799	NI	16.9±12.5 mmol/L	NI	2005–2011	Didn´t include control group.
Moghaddasi [49]	2010	Iran	Retro	Case-control	HPLC	82	60	21.1 (9.8) mmol/L	13.5 (3.2) mmol/L	2009	NI
Yuetao [70]	2010	China	Prosp	Case-cohort	HPLC	377	106	21.0 (12.8) mmol/L	18.7 (11.2) mmol/L	2007–2008	Used TIA patients as group control
Yan [65]	2017	China	Prosp	Case-series	standard laboratory methods	238	NI	16.0 (11.2–23.4) mmol/L	NI	2014–2015	Compared IS patients with and without depression.
Kelly [71]	2002	United States	Retro	Meta-analysis	NI	1487	2554	13.51 mol/L	11.07 mol/L	1966–2001	Didn´t include SD
Ashjazadeh [72]	2013	Iran	Retro	Case-control	axis homocysteine enzyme immunoassay	171	86	16.2 mmol/L (14.8 to 17.5)	13.5 mmol/L (12.4 to 14.6)	2009–2010	NI
Haapaniemia [73]	2006	Finland	Prosp	Case-series	standard competitive immunoassay method	102	102	9.6 (11.4 ± 6.5) mmol/L	11.6 (13.7 ± 7.1) mmol/L	2006	Hct was significantly lower in patients than in controls on admission
Niaz [74]	2019	Pakistan	Prosp	Case-control	NI	71	NI	22.76±12.67 mmol/L	NI	2017–2019	Patients younger than 45 years old.
Jingjuan [75]	2017	China	Retro	Case-control	enzymatic cycling assay	987	NI	13.9 ± 6.5 mmol/L	NI	2011–2014	NI
Shan shan [67]	2018	China	Retro	Case-control	HPLC	75	19	16.78±5.52 mmol/L	(13.63±5.01) mmol/L	2016–2017	Control group constituted of patients with atheromatous plaques in brain arteries, without IS.
Ling-Cong [53]	2017	China	Retro	Case-control	NI	120	NI	27.57±20.17 mmol/L	NI	2011–2014	NI
Kawamoto [58]	2002	Japan	Retro	cross-sectional	HPLC	44	47	14.6±5.6 mmol/L	12.6±6.6 mmol/L	2000	NI
Ying-Li [59]	2018	China	Prosp	Case-series	enzymatic methods using test kits	189	NI	11.90 (9.20–14.85) mmol/L	NI	2013	NI
Gungor [76]	2018	Turkey	Retro	Case-control	HPLC	262	NI	high (≥16 mol/L) in 99 patients (37.79%). Homocysteine level was normal (≤14 mol/L) in 163 patients (62.21%).	NI	2006–2016	NI
Biswas [77]	2009	India	Prosp	Case-series	EIA	120	120	12 (5.3–30.1) mmol/L	11.2 (6.2–14.2) mmol/L	2003–2007	NI
Macko [78]	2002	Norway	Prosp	Case-series	HPLC	49	NI	11.3 (±2.8 mmol/L)	NI	2001	NI
Chang-yi [68]	2014	China	Retro	cross-sectional	automatic biochemical analyzer	111	2817	17.3 (13.1–25.7) mmol/L	14.5 (12.0–18.5) mmol/L	2010–2011	NI
Rudreshkumar [9]	2017	India	Prosp	Case-series	HPLC	171	249	20.79 ± 1.77 mmol/L	11.10 ± 0.23 μmol/L	2014–2016	NI
Kalita [79]	2009	India	Prosp	Case-series	NI	198	200	19.99 ± 12.77 mmol/L	18.41 ±12.01 μmol/L	2004–2006	NI
Mizrahi [32]	2005	Israel	Retro	Case-control	HPLC	113	NI	12.99 ± 7.17 mmol/L	NI	2004	NI
Sawula [60]	2009	Poland	Retro	Case-control	HPLC	131	64	16.8 mmol/L	10.4 mmol/L	2007	NI
In-Uk [61]	2008	South Korea	Prosp	Case-series	NI	267	76	16.29 ±8.3 mg/dL	12.53 (±3.77) mg/dL	2006–2007	NI
Dhamija [80]	2009	India	Retro	Case-control	ELISA	66	72	28.40 ±2.08 mmol/L	11.16 ±1.09 mmol/L	2008	NI
Kelly [33]	2004	United States	Prosp	Case-series	NI	156	118	10.1 mmol/L	10.33 mmol/L	2003	NI
Zhaohui [14]	2003	China	Retro	Case-control	HPLC	1823	1832	14.7 mmol/L	12.8 mmol/L	2000–2001	NI
Verhoef [43]	1994	United States	Retro	Case-control	HPLC	109	427	11.1±4.0 nmol/mL	10.6±3.4 nmol/mL	1982–1984	NI
Beynum [66]	1999	The Netherlands	Retro	Case-control	HPLC	45	234	8.5 mmol/L (range, 5.0 to 77 mmol/L)	9.1 mmol/L (range, 4.3 to 20.0 mmol/L)	1989–1997	IS in patients from 0 to 18 yeas old.
Guan-Hui [81]	2017	China	Prosp	Case-series	automated biochemistry analyzer	86	182	17.45 (15.10–21.28)	14.45 (12.35–16.60)	2012–2015	NI

Ischemic Stroke (IS), homocystein (Hct), standard deviation (SD), increased homocysteine (iHct), Retrospective (Retro), Prospective (Prosp), Glasgow Coma Scale (GCS), Fluorescence polarization immunoassay (FPA), highperformance liquid chromatography (HPLC), Not informed (NI), Internal Carotid (IC), Statistic Significance (SS), transient ischemic attack (TIA).

### Data collection, data extraction, and quality assessment

Two researchers independently extracted demographic data, number of cases and controls, Hct level and corresponding dispersion measures (standard deviations or confidence intervals). The NOS was used to perform quality assessment. The control population used by the individual studies was analyzed to avoid comparison with other cerebrovascular diseases [14–17].

### Statistical analysis and literature search

We employed a random-effects inverse-variance weighted meta-analytical approach in order to pool standardized mean differences, with estimation of τ^2^ through the DerSimonian-Laird method. Due to the large heterogeneity, random-effects models were used to calculate the standardized mean difference (SMD) with 95% confidence intervals (CI). Standard error funnel plots were used to assess publication bias, and sensitivity analyses were performed to evaluate the results after removing potential outliers. All analyses were performed using R (*R Foundation for Statistical Computing*. Vienna, Austria, 2018).

## Results

### Systematic review

The initial search yielded 1361 studies (Fig 1). Studies were categorized as either prospective or retrospective if the blood sample used to assess Hct was collected before or after the stroke event respectively. After careful analysis of abstracts and full-texts, the meta-analysis included data from 38 studies, which involved almost 16 000 stroke events [8, 25–31].

Data for all 38 studies regarding year of publication, country, study design, method for detecting homocysteine, number of patients, mean homocysteine in each group, enrollment period and main finding is presented in Table 1. However, only 13 studies reported means and standard deviations for cases and controls, and therefore were used in the meta-analysis. Those 13 studies presented data from 5002 patients with stroke and 4945 controls.

### Meta-analysis

**Fig 2:**
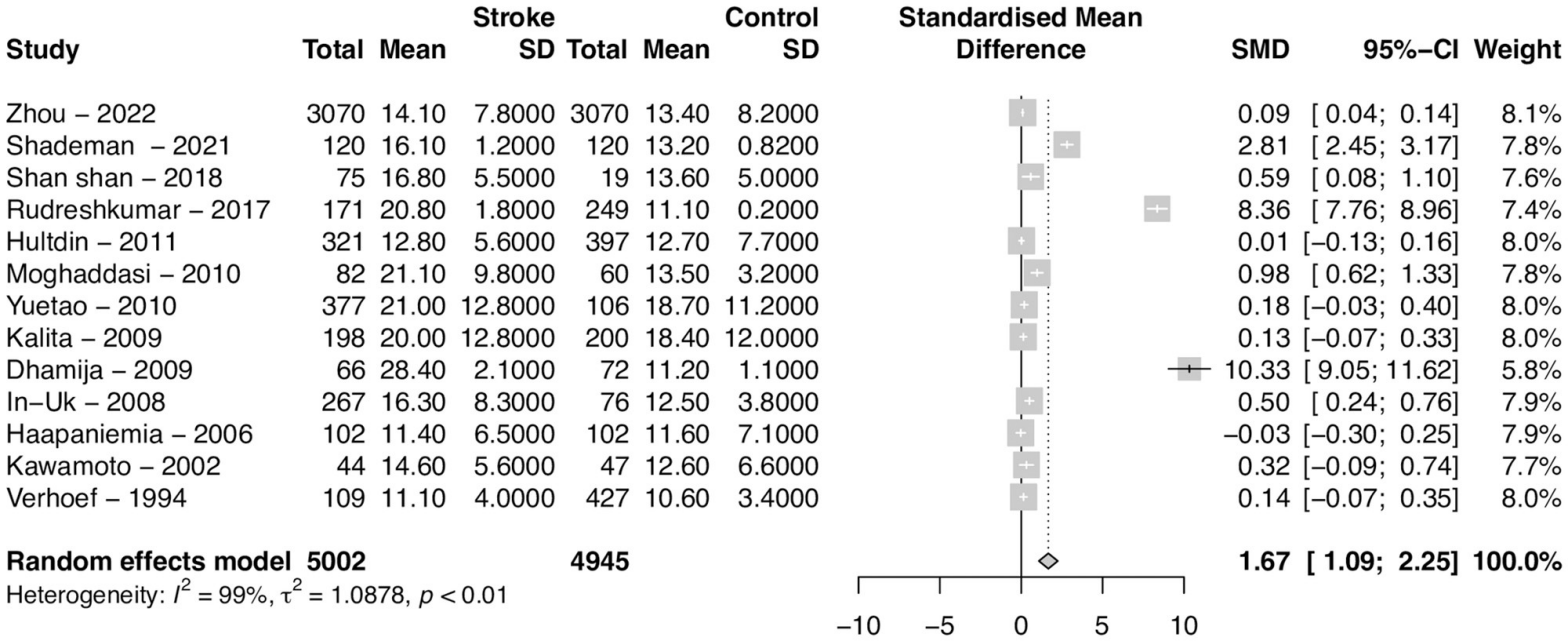
Difference in homocysteine levels between patients with ischemic stroke and controls. Forest plot of the standardized mean difference of homocysteine levels between patients with stroke and controls. Cases had homocysteine levels 1.67 mol/L higher than controls (95%CI 1.09–2.25). Between-study heterogeneity was very large, as demonstrated numerically by the heterogeneity measure R = 99%.

The results of the meta-analysis are shown in Fig 2. The standardized mean difference was 1.67 (95% CI 1.09–2.25, p < 0.01), indicating that homocysteine levels were significantly higher in patients with ischemic stroke compared to controls. Between-study heterogeneity was very large (*I*^*2*^ = 99%), particularly because two studies showed significantly large mean differences.

### Sensitivity analysis

**Fig 3:**
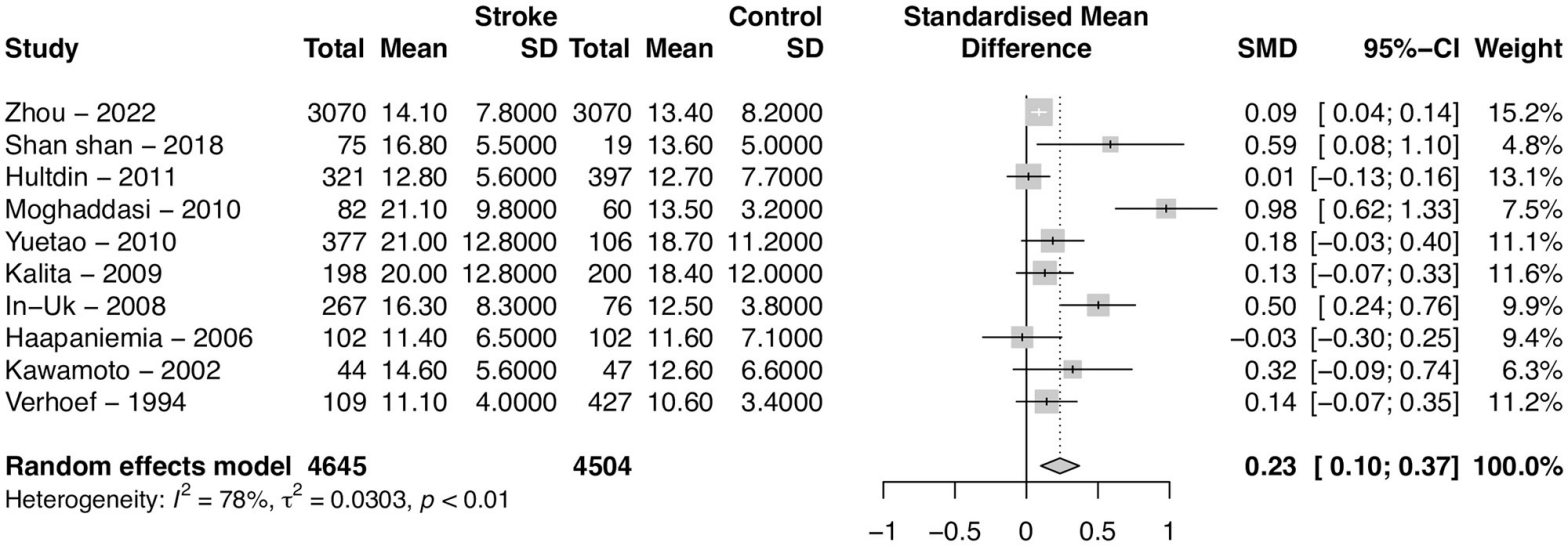
Sensitivity analysis excluding outliers. Forest plot of the Standardized Mean Difference (SMD) of homocysteine levels between patients with stroke and controls after removal of two discrepant studies. Cases had higher levels of homocysteine compared to controls. The SMD is still statistically significant, albeit smaller (0.23 umol/L, 95% CI 0.10–0.37). Heterogeneity decreases by 21 percentage points but is still significant (R = 78%).

Sensitivity analysis was performed to assess the results if those three outliers were to be discarded, as shown in Fig 3. When excluding Dhamija (2009), Rudreshkumar (2018) and Shademan (2021), we are left with 4645 cases and 4504 controls. The results show a significant difference, albeit much smaller (SMD = 0.23, 95% CI 0.1–0.37, p = 0.0006), and an important decrease in heterogeneity (*I*^2^ = 78%).

### Publication bias

**Fig 4:**
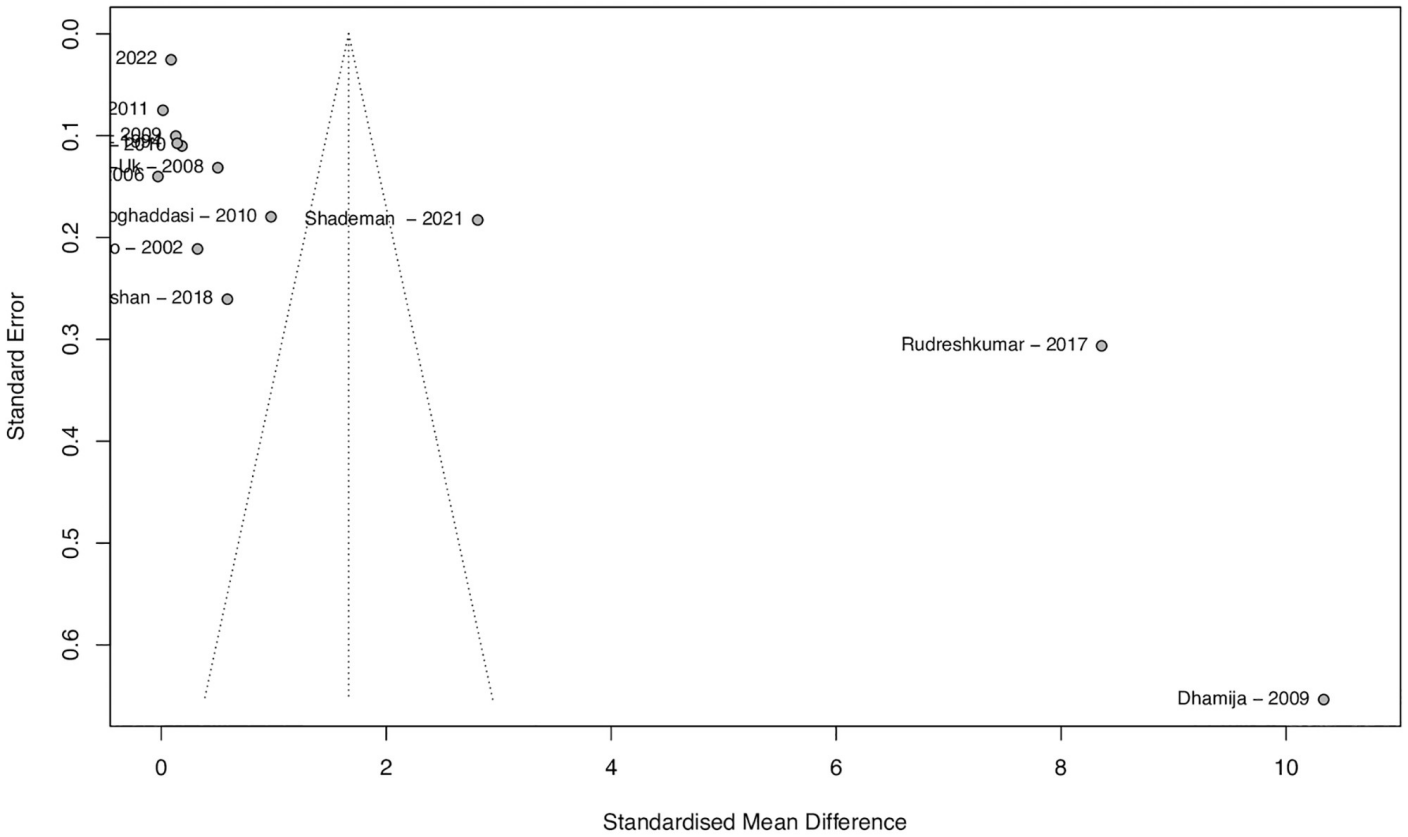
Funnel plot for assessment of publication bias. Funnel plot for assessment of publication bias, in which three outliers are identified in the far-right hand of the abscissa (Rudreshkumar et al., Dhamija et al. and Shademan et al.). The large heterogeneity among studies is demonstrated graphically.

**Fig 5:**
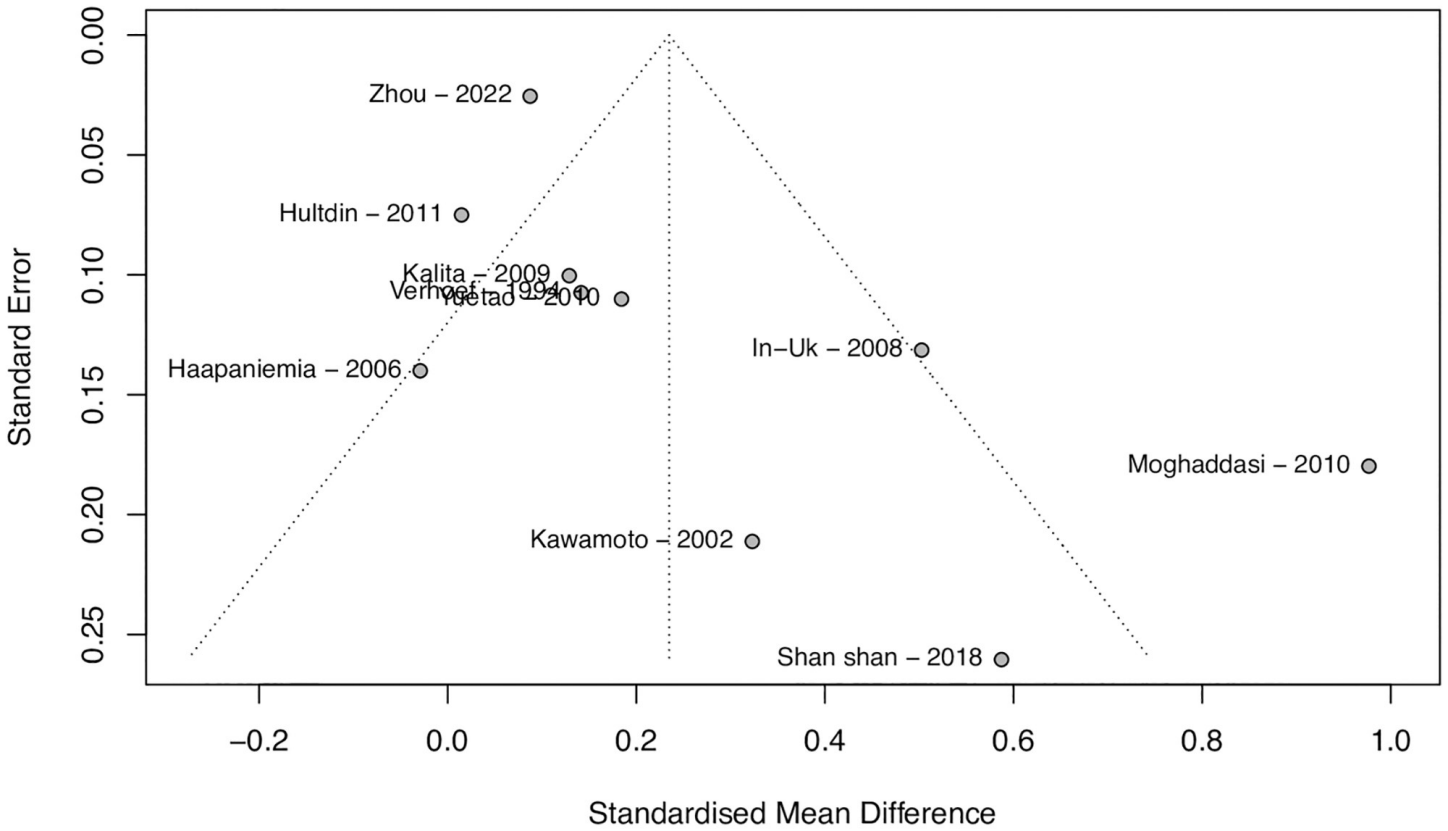
Funnel plot for assessment of publication bias in the sensitivity analysis. Funnel plot for assessment of publication bias in the sensitivity analysis, after removal of the outliers. Although heterogeneity is still large, the distribution is more balanced, and there is no obvious evidence of publication bias.

The funnel plot in Fig 4 shows an unbalanced variance distribution because of the three outliers previously mentioned: Dhamija et al. (2009) and Shademan (2021) compared serum Hct between patients with ischemic stroke and healthy controls; and Rudreshkumar et al. (2018), compared young patients with ischemic stroke and healthy controls [26, 32, 33]. The funnel plot for the sensitivity analysis is shown in Fig 5, demonstrating a much more balanced assessment regarding publication bias.

### Subgroup analysis—Prospective studies

**Fig 6:**
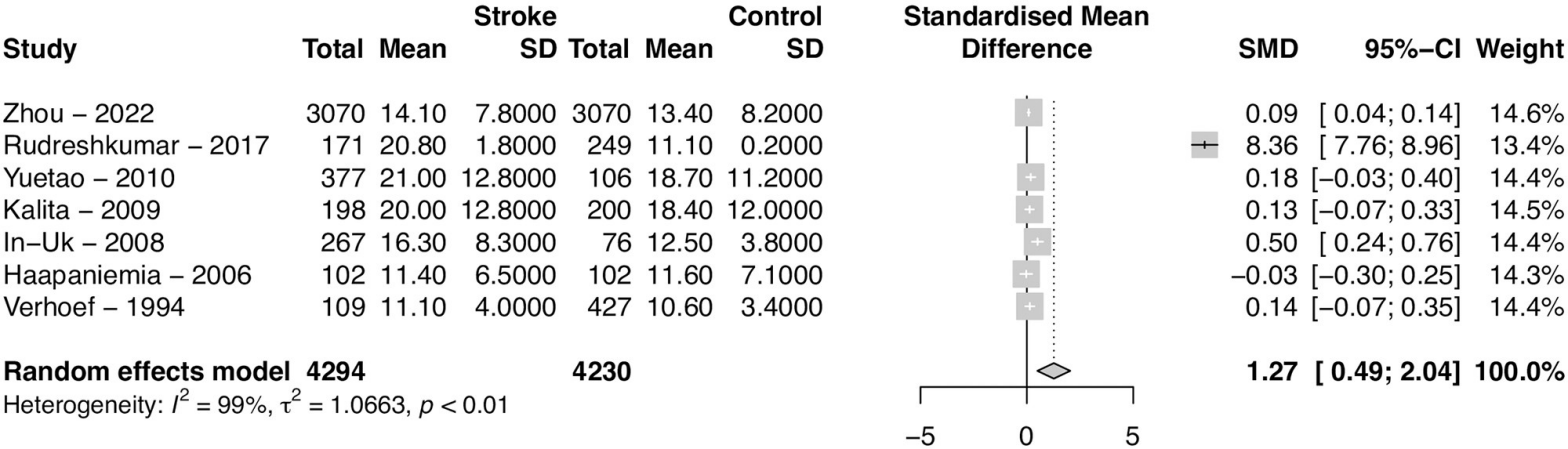
Prospective studies. Forest plot of the Standardized Mean Difference (SMD) of homocysteine levels between patients with stroke and controls for prospective studies. Hct levels were significantly higher in stroke patients (SMD = 1.27, 95% CI 0.49–2.04, *p = 0*.*0013*). Heterogeneity was very large (*I*^2^ = 99%).

There were seven prospective studies detailing Hct levels for stroke and control patients. Analyzing those studies separately, Hct levels were significantly higher in stroke patients (SMD = 1.27, 95% CI 0.49–2.04, *p = 0*.*0013*). The heterogeneity was very high (*I*^2^ = 99%) (Fig 6).

### Subgroup analysis—Retrospective studies

**Fig 7:**
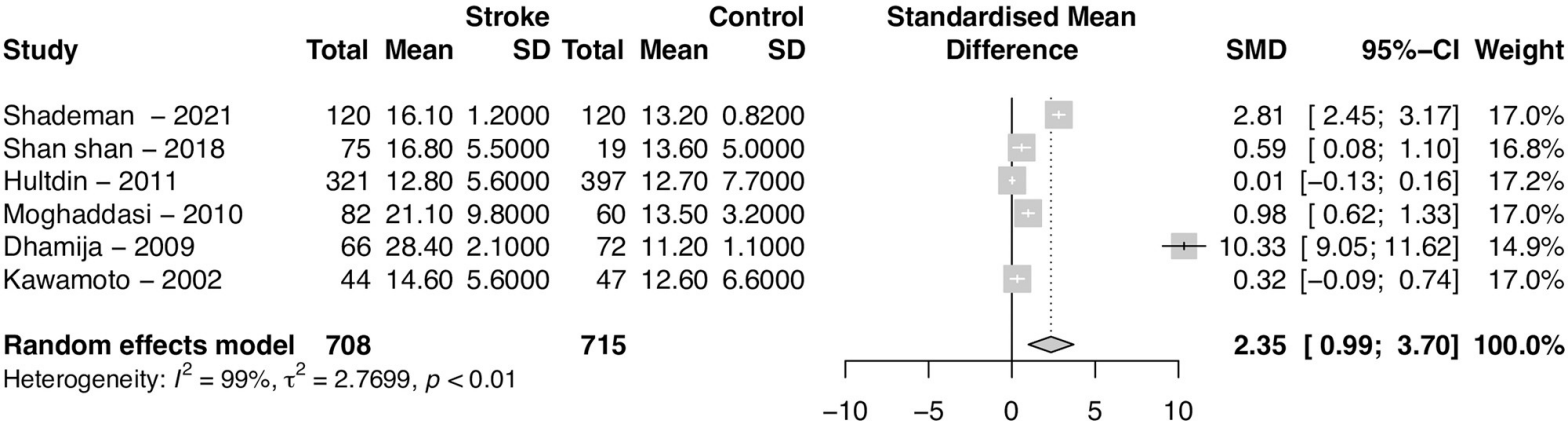
Retrospective studies. Forest plot of the Standardized Mean Difference (SMD) of homocysteine levels between patients with stroke and controls for retrospective studies. Hct levels were significantly higher in stroke patients (SMD = 2.35, 95% CI 0.99–3.70, p = *0*.*0007*). Heterogeneity was also very large (*I*^2^ = 99%).

There were six retrospective studies detailing Hct levels for stroke and control patients. Analyzing those studies separately, Hct levels were also significantly higher in stroke patients (SMD = 2.35, 95% CI 0.99–3.70, p = *0*.*0007*). The heterogeneity was also very large (*I*^2^ = 99%) (Fig 7).

### Subgroup analysis—TOAST stroke subtypes

**Fig 8:**
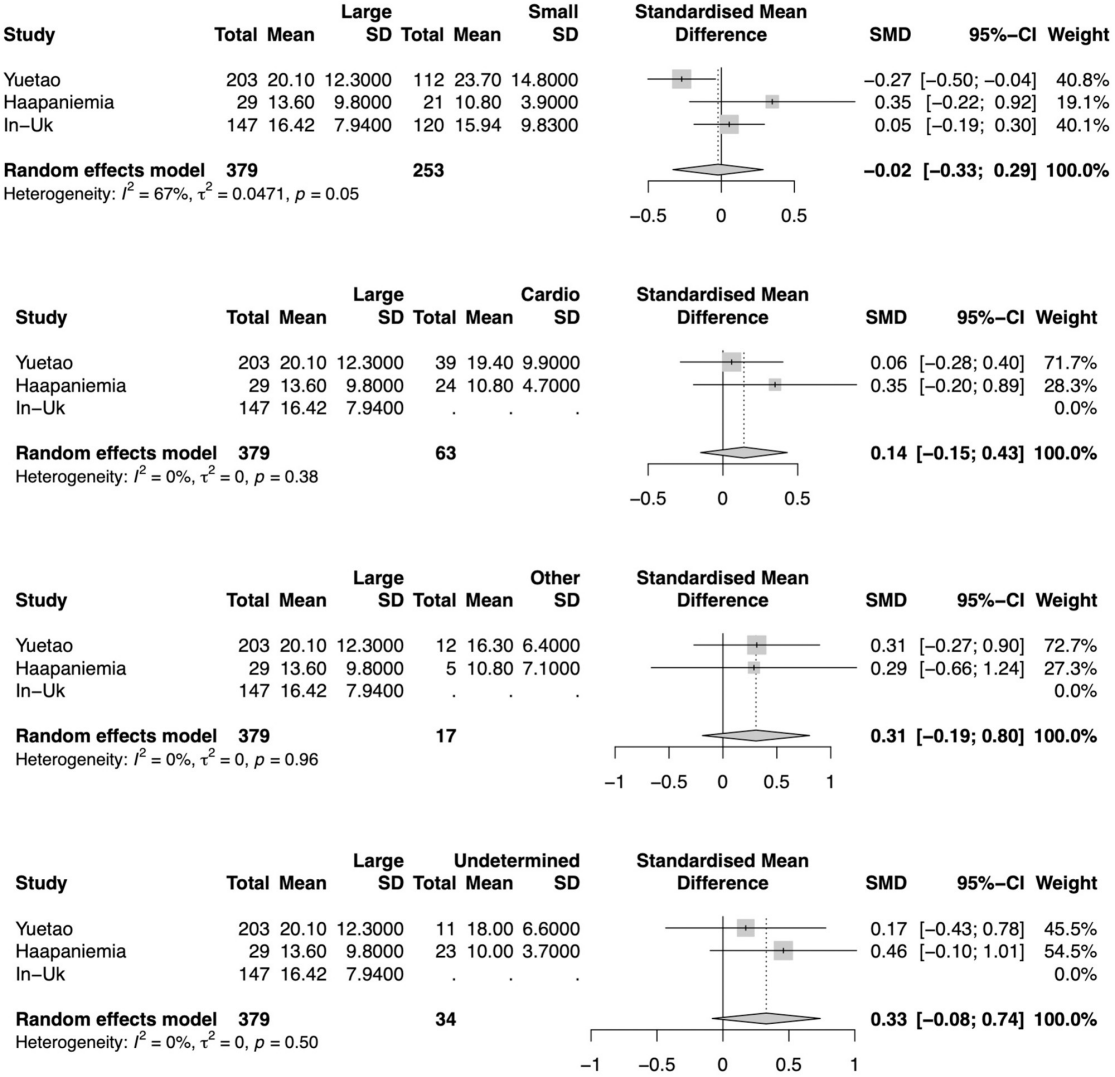
Difference in homocysteine levels for each stroke subtypes. Forest plot of the Standardized Mean Difference (SMD) of homocysteine levels between patients for each stroke subtype and controls. Homocysteine levels were not significantly different for any subtype.

There were three studies detailing Hct levels for stroke and their subtypes. Analyzing those studies separately, Hct levels were not significantly different in any subtype patients. Heterogeneity was also very large (*I*^2^ = 67%) for the large-artery atherosclerosis and small-vessel occlusion comparison. All other comparisons had a small heterogeneity (*I*^2^ = 0%) (Fig 8).

**Fig 9:**
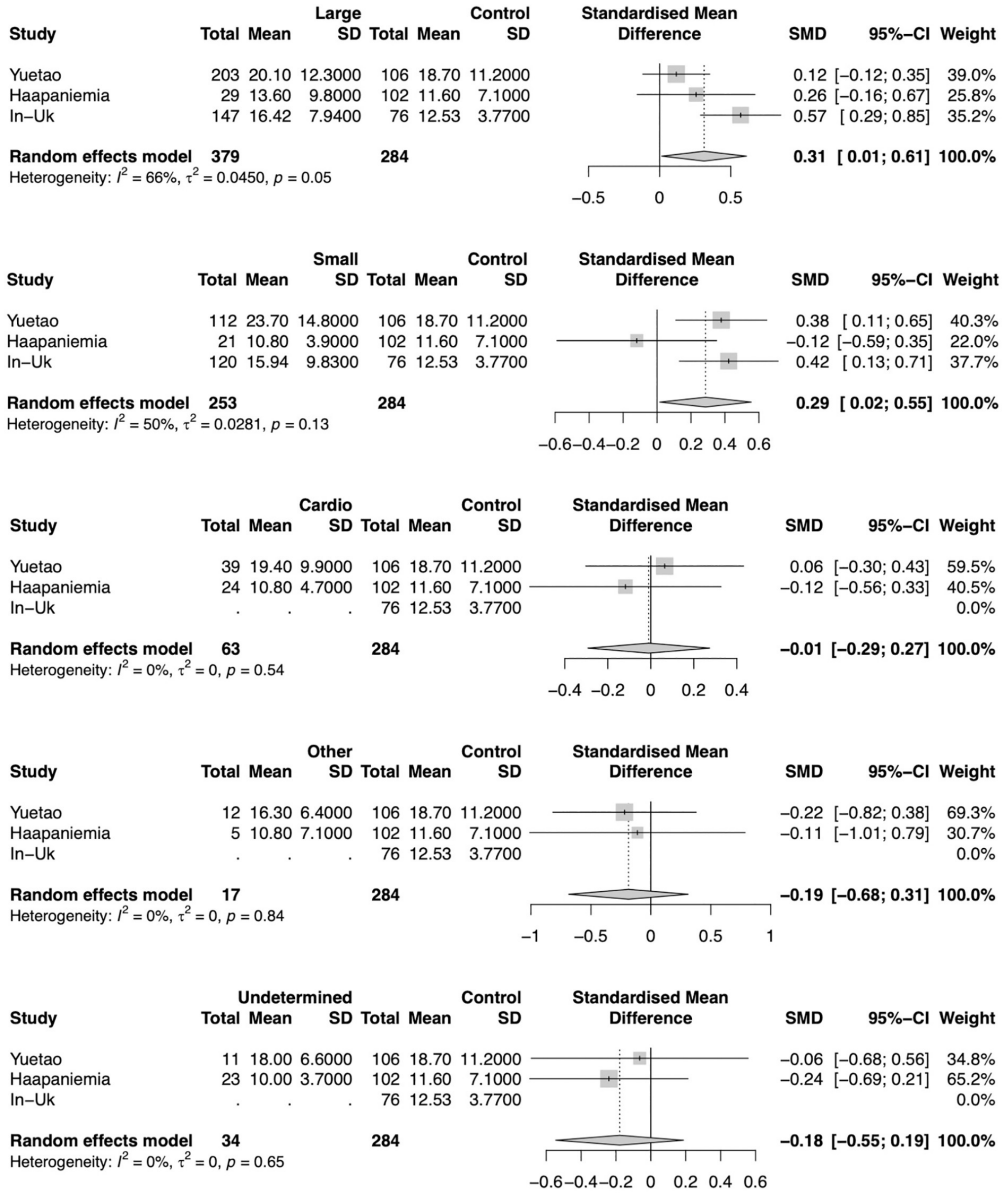
Difference in homocysteine levels for ischemic stroke subtypes and controls patients. Forest plot of the Standardized Mean Difference (SMD) of homocysteine levels between patients for each stroke subtype and controls. Patients with large-artery atherosclerosis (SMD = 0.31, 95% CI 0.01–0.61; p = 0.0403) and small-vessel occlusion (SMD = 0.29, 95% CI 0.02–055; p = 0.0379) had significantly higher homocysteine levels than control patients.

When comparing each stroke subtype with control patients, large-artery atherosclerosis (SMD = 0.31, 95% CI 0.01–0.61; p = 0.0403) and small-vessel occlusion (SMD = 0.29, 95% CI 0.02–055; p = 0.0379) were significantly higher in stroke patients. The heterogeneity was also very large for comparison of large-artery atherosclerosis (*I*^2^ = 66%) and small-vessel occlusion (*I*^2^ = 50%). All other comparisons had a small heterogeneity (*I*^2^ = 0%) (Fig 9).

## Discussion

Stroke is emerging as an increasing public health problem, especially due to long-term disability among patients. Early detection and intervention represent the best way to protect against stroke complications. Nevertheless, there is no biomarker thus far that may correlate with stroke. High level of Hct has been found in atherosclerosis patients. Previous *in vivo* and *in vitro* studies suggest that Hct changes the endothelium to a thrombogenic phenotype. Therefore, Hct thus has adverse effects on endothelial function, stimulates smooth muscle proliferation, and is a procoagulant, thus increasing the risk of major vascular events [10, 11, 33–49].

Patients with ischemic stroke disease have higher Hct levels than controls. Three studies identified large differences [26, 32, 33] but the vast majority of them demonstrated a tendency of higher levels of Hct in patients with stroke compared to controls, which was confirmed in the sensitivity analysis that excluded those three outliers. [9–11, 34, 50].

Dai D et al. [51] establish that inverse association between Glasgow Coma Scale and Hct concentrations among patients with hemorrhagic stroke, especially in ever smokers, or in participants with higher systolic blood pressure or total cholesterol levels. Hct level may be an aggravating factor in atherosclerosis, which is positively associated with a high risk of stroke [51–57].

It is highly likely that iHct can be regarded as an aggravating factor that elevates the risk of ischemic stroke, since it triggers the necrosis of vessel walls and pathophysiology of endothelial degeneration. It can interfere with the stability of the blood-brain barrier through an excitotoxicity pathway related to NMDA channels, and it can decrease the activity of numerous essential cellular components such as Na/K ATPase, superoxide dismutase, and glutathione peroxidase [32, 58, 59].

Hct activates the transcription of the factor in nervous tissue, which increases inflammation by increasing the levels of inflammatory cytokines. The role of this molecule in epilepsy, anxiety, cardiovascular diseases and stroke leaves no doubt that it could be a key aspect of ischemic stroke pathogenesis [55, 56, 60, 61].

Hyperhomocysteinemia is currently recognized as a risk factor for ischemic stroke and human vascular disorders. Nonetheless, it is very imprecise to determine if iHct triggers the pathogenesis of the diseases or it signifies a biomarker of metabolic aberrations. Hyperhomocysteinemia changes how vascular endothelial cells function as well as activating homocysteinylation and thiolation of enzymes and plasma proteins which negatively affect the brain parenchyma and cerebrovascular. Under iHct conditions, ischemic reperfusion injury usually triggers degeneration processes as well as engage in deregulation of intracellular signaling.

Moreover, Acampa et al. [62] showed that iHct can favor atrial cardiopathy and silent episodes of atrial fibrillation occurrence with multiple mechanisms: direct effects on atrial ionic channels (electrical remodeling); biochemical damage on atrial extracellular matrix (structural remodeling); prothrombotic state, favoring atrial thrombosis and possible subsequent ischemic embolic stroke. Another study by Acampa et al. [63] also shows that iHct is associated with an alteration in electrical atrial conduction, possibly contributing, at least in part, to the increased risk of cardiac arrhythmias and, therefore, a risk factor for stroke.

Yan Ji et al. [64] demonstrated that hyperhomocysteine treatment with B vitamin supplementation significantly reduced stroke events. Factors including the folate fortification of cereal products, follow-uptime, status of absorption and response to B vitamin supplementation, the existence of chronic kidney disease or high blood pressure, and the use of medication can influence the effects of B vitamin supplementation.

Shi et al. [47] observed that high plasma Hct levels in the acute ischemic stroke are strong mortality predictors in patients with severe atherosclerosis. There is a relevant, independent association with high Hct levels and the risk of mortality from patients affected with ischemic stroke. Weikert C et al. [46] suggest that vitamin B12 low plasma levels, in combination with folate low levels, probably increase the risk of ischemic Stroke. This effect may be mediated at least partly through elevations of homocysteine levels. Kwon et al. [45] proposed that high Hct increase the risk of early neurological deterioration.

It has been proven that the elevation of plasma Hct is often correlated with the development of atherosclerosis as well as the impairment of the vascular endothelium. Additionally, it triggers serine elastase synthesis in the vascular smooth muscle cells leading to elastolysis by degrading the extracellular matrix and releasing reactive oxygen species.

Fan et al. described that Hct and arterial hypertension independently correlated with moderate/severe neurological severity in stroke patients and this combination make significant correlation of interaction on neurological severity [59], and depression 3-months after stroke [65]. Shi Z et al. [47]. Demonstrate that iHct levels during the first 24 hours after admission for an acute stoke significantly mortality in 48 hours.

van Beynum et al. reported an association between occlusive vascular and stroke disease and hypehomocysteinemia [66]. In a prospective study was observed 20% higher risk of ischemic stroke in men associated with homocysteine values. High plasma Hct levels are associated with intracranial strong plaque and carotid plaque and intima-media thickness [67].

Chang-yi et al. [68] described that renal impairment was associated with a more advanced age onset, a higher frequency of previous stroke and hypertension, a higher NIHSS score at admission, lower HDL levels, and iHct compared to normal real function [68]. Renal insufficiency is a risk factor of cerebrocardiovascular events and indicates poor prognosis in many patients.

Zhang et al. (2020) reported in their metanalysis the association of iHct blood levels with acute stroke treatment patients (2243 patients) compared with controlled group (871 patients) and showed that the subtypes “small-vessel occlusion” and “large-artery atherosclerosis” are the one most associated with iHct. However this study represented just Chinese population with few patients included [10]. Our study showed similar results, with those two subtypes having significantly higher levels of iHct compared to control patients. There was no significant difference when comparing iHct levels between small-vessel and large artery subtypes.

A metanalysis performed by Homocysteine Studies Collaboration in 2002, with 30 prospective and retrospective papers, but only 1113 stroke events, concluded that elevated homocysteine is a modest independent predictor of IHD and stroke risk in healthy populations [48]. Nevertheless, Wu et al. and He Y [11, 69], demonstrated also in their metanalysis the relation between stroke events and iHct, but do not distinguish between ischemic and hemorrhagic strokes and few patients were analyzed.

Huang S et al., recently published a metanalysis showing a correlating between high levels of Hct and gender, B12 deficiency, smoking and patients who received tissue plasminogen activator treatment, but no significant differences related to age, drinking, hypertension, diabetes mellitus o hyperlipidemia in patients with stroke. However, the authors did not specify which sex, level of B12, and smoking status are correlated with Hct, decreasing the quality of the study. They also suggest that Vitamin B supplementation has a significant protective effect on recent stoke, and high levels can also predict prognosis and suggest that thrombolytic therapy may be helpful in the high-risk group. Nevertheless, they have established an arbitrary cut-off of 20 μmol/L, even the results of their analysis founded that high level of 18.6 μmol/L had 1.61 increased risk of death. This bias was not clear. This cut-off contrast with our findings because the studies analysis data shows that there were too much heterogenous with different population. Therefore, it is necessary more robust studies to reliably determine a cut-off [69].

Our meta-analysis has many strengths. To our knowledge, this is the first study that provides a comprehensive systematic review and meta-analysis of Hct levels and ischemic stroke disease, including just ischemic stroke and a large amount of data. The study involved almost 16 000 stroke events and 38 prospective and retrospective studies.

However, there are still several limitations applied to this meta-analysis, for instance, different studies especially those who adopted the retrospective design might be subjected to artifact and bias as compared to the prospective studies. A significant heterogeneity, as described in the analysis, should also prompt careful interpretation of the results. Furthermore, there is a probability that the journals published only positive findings since the meta-analysis solely used published data. Additionally, reliability and sensitivity issues may have been related to the diverse detection methods used for elevated plasma Hct. Another important limitation is regarding the lack of data in the studies about the follow up duration.

The cross-sectional design makes it difficult to establish if the altered parameters are causally linked to stroke. Even though many of the included papers state that Hct is a risk factor for stroke, the studies performed so far do not allow this conclusion, by design, since all of them analyze a population that has already had the acute event. It is not possible, to date, to establish a cutoff for serum Hct that would confer greater risk of stroke or other cerebrovascular diseases. Ideally, larger prospective cohorts are necessary to confirm the cause-effect relationship. Additionally, since the researchers only included papers published in English, there is a high probability that some relevant studies might have been excluded.

## Conclusion

This meta-analysis demonstrates that patients with stroke have higher levels of Hct compared to controls. Whether this is a modifiable risk factor remains to be assessed through larger prospective cohorts. Approaching modifiable risk factors of ischemic stroke is paramount since these are leading causes of mortality and morbidity worldwide.

## Supporting information

S1 Checklist PRISMA 2020 checklist. (DOCX)
